# Establishing company level fishing revenue and profit losses from fisheries: A bottom-up approach

**DOI:** 10.1371/journal.pone.0207768

**Published:** 2018-11-20

**Authors:** Tim Cashion, Santiago de la Puente, Dyhia Belhabib, Daniel Pauly, Dirk Zeller, U. Rashid Sumaila

**Affiliations:** 1 *Sea Around Us*, Global Fisheries Cluster, Institute for the Oceans and Fisheries, University of British Columbia, Vancouver, British Columbia, Canada; 2 Fisheries Economics Research Unit, Global Fisheries Cluster, Institute for the Oceans and Fisheries, University of British Columbia, Vancouver, British Columbia, Canada; 3 Fish Tracker Initiative, London, United Kingdom; 4 Quantitative Modeling Group, Institute for the Oceans and Fisheries, University of British Columbia, Vancouver, British Columbia, Canada; 5 Ecotrust Canada, Vancouver, British Columbia, Canada; 6 *Sea Around Us* – Indian Ocean, School of Biological Sciences, University of Western Australia, Crawley, Western Australia, Australia; Aristotle University of Thessaloniki, GREECE

## Abstract

A third of global fish stocks are overexploited and many are economically underperforming, resulting in potential unrealized net economic benefits of USD 51 to 83 billion annually. However, this aggregate view, while useful for global policy discussion, may obscure the view for those actors who engage at a regional level. Therefore, we develop a method to associate large companies with their fishing operations and evaluate the biological sustainability of these operations. We link current fish biomass levels and landings to the revenue streams of the companies under study to compute potentially unrealized fisheries revenues and profits at the level of individual firms. We illustrate our method using two case studies: anchoveta (*Engraulis ringens;* Engraulidae) in Peru and menhaden in the USA (*Brevoortia patronus* and *B*. *tyrannus;* Clupeidae). We demonstrate that both these fisheries could potentially increase their revenues compared to the current levels of exploitation. We estimate the net but unrealized fishery benefits for the companies under question. This information could be useful to investors and business owners who might want to be aware of the actual fisheries performance options of the companies they invest in.

## 1.1 Introduction

The marine fishing industry is currently failing to realize economic benefits estimated to be between USD 51 and 83 billion annually [1–3]. These unrealized benefits are largely due to stocks whose biomass has been reduced below levels that generate maximum sustainable yields (MSY), which leads to their continued exploitation generating higher costs. While some regions are experiencing a growth in fish catches due to rebuilding fish stocks [4], global catches are declining at a rate of 1.2 million tonnes annually [5]. These current trends illustrate the global picture of the state of the oceans, and point to the lost economic potential of capture fisheries. However, it also presents a major opportunity and incentives to rebuilding stocks globally. Through strong rebuilding, the benefits of increased catches and lower costs of fishing could outweigh short-term costs of temporarily reduced catches within a decade or so [2].

A major challenge to addressing these potentially unrealized profits at the global scale is the opacity of seafood supply chains. Seafood is among the most highly traded food commodities [6], but its supply chains often include illegal and unreported fish or fish that is mis-labelled [7,8]. Thus, the stock-origin of much of the globally traded seafood supply is not readily ascertainable. Unreported and especially illegal fisheries production can be implicated in unsustainable fisheries practices, and creates challenges for the proper management of resources. Therefore, one major obstacle to separating sustainably and unsustainably caught seafood is the lack of transparency and traceability in industry supply chains.

In response to concerns about overfishing and the lack of transparency, as well as the general state of marine fisheries, many certification, ecolabeling and traceability schemes have been proposed, each with their own criteria of what makes a fishery or aquaculture system ‘sustainable’. For fisheries, the largest ecocertification initiative is the Marine Stewardship Council, which now covers 14% of reported global landings, and is aiming for 33% by 2030 [9]. However, this will require voluntary buy-in from the fishing companies involved, thus limiting the scope of these certification schemes’ effectiveness on the industry outside their program. In addition, the adequacy and enforcement of ecocertification criteria have come under scrutiny [10,11]. For example, the *Star Shrimper XXV* is certified as a sustainable prawn fishing vessel under the ‘Friend of the Sea’ criteria because it uses nets that reduce turtle bycatch; however, the vessel was detained for fishing in the Exclusive Economic Zone (EEZ) of Liberia without an access agreement and while not using their selective fishing gear [12].

One alternative to ecocertification and labeling schemes is an external review that links fishing practices of specific fish stocks to individual fishing companies, to better inform owners and potential investors. To gain a better coverage of the fishery practices of the seafood industry, we take this latter approach with regards to large seafood companies some of which are already involved in other seafood sustainability initiatives outside ecocertification programs [13]. Previous external reviews of fishing companies have demonstrated company links to illegal fishing, in addition to human trafficking and other illicit practices [14]. Clearly there are reputational and legal risks to these companies engaged in these activities, but here we focus on the biological sustainability of the fisheries they are engaged in and its implications for their economic returns.

Our objective is to link specifically identified fisheries stocks with declining catches to the companies that are the key actors in those particular fisheries. Thereby, we aim to demonstrate the potential economic losses, or unrealized revenues and profits, at the specific company level from fisheries targeting overfished stocks, or not realizing the full potential benefits of currently lightly fished stocks.

We have developed an approach that attempts to help clarify the opaque environment that characterizes many seafood supply chains. Through a diversity of methods, we characterize current fishing practices of companies and relate a company’s products to the fishing grounds and the fish stocks targeted. We then evaluate the changing revenue streams from these fisheries due to changing fish populations and evaluate the fishery stock-status using established catch-based indicators of stock health applicable to every fishery in the world. It is worth noting that in current application, we explore linkages at the origins of the seafood supply chains (i.e., fish in the ocean with the companies that catch them). However, this method could be easily extended to processors and sellers downstream; hence encompasing complete seafood supply chains. We acknowledge the uncertainties linked to the latter approach, although similar uncertainties exist in other stock assessment approaches. However, the data we use reflect a collection of industry, national, reconstructed, and peer-reviewed data that together create a reasonably complete picture of these fisheries.

## 2.1 Methods

**Fig 1:**
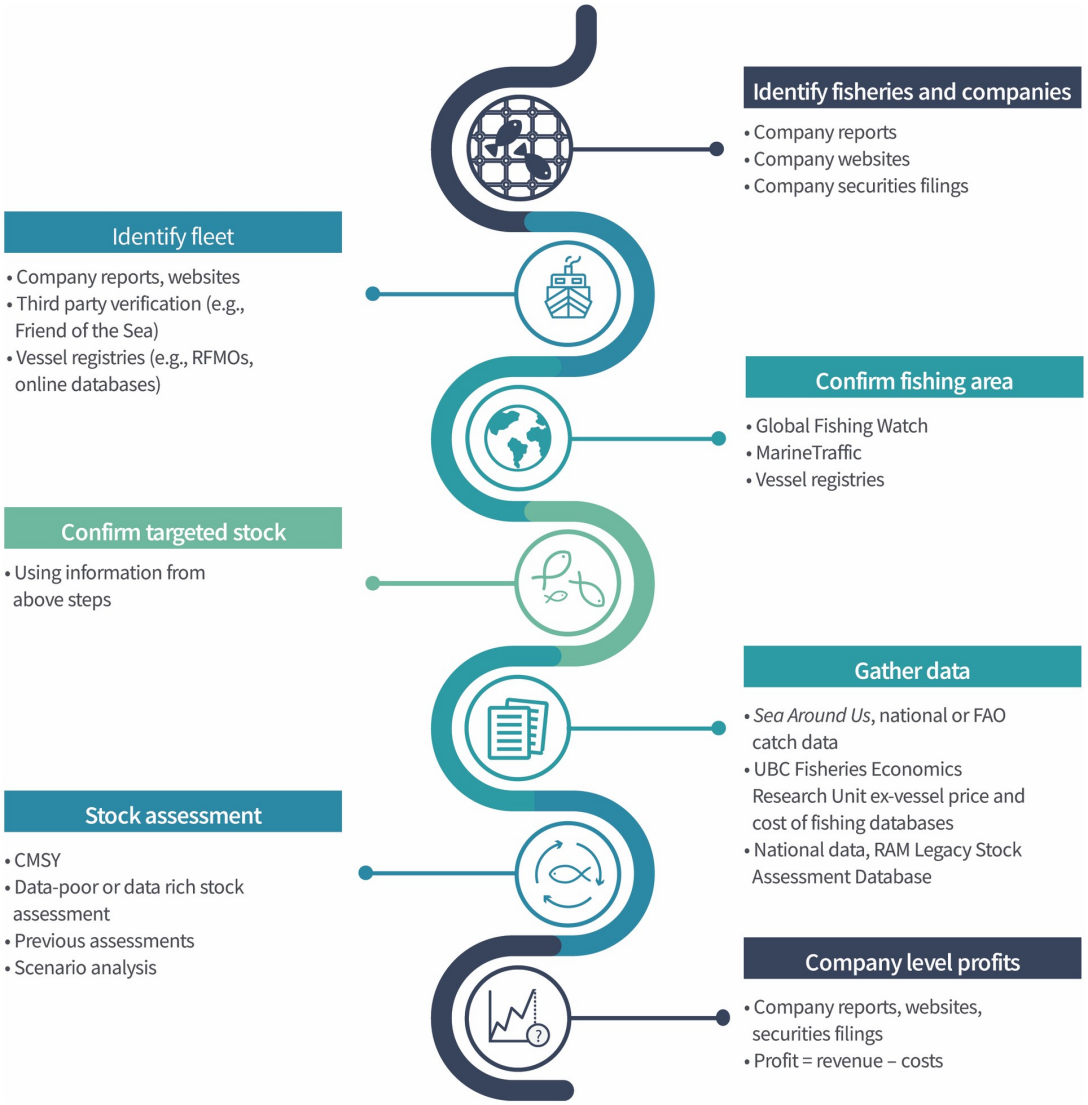
Schematic representation of method for linking fish stocks to company level fisheries revenues and unrealized profits.

We developed a seven-step method to link any fishing company (including its revenues and profits) with the fish resources that it exploits and their biological status (Fig 1). While we outline this here as a step-by-step process for clarity, in reality it can be iteratively reevaluated as more information becomes available from different sources. In addition, there is often uncertainty with regard to the actual resources companies exploit (i.e., which specific stock of which specific species in which specific area), largely due to non-clarity in company reporting. The steps we provide can be adapted, whether a specific set of resources or a specific company is the focus of the study. Companies that are publicly traded on stock exchanges are more amenable to this method, as they have legal requirements to disclose information for shareholders, which makes assigning their landings generally more straightforward and less uncertain than for private companies.

***Step 1*** is to choose a fishery of interest and to identify the companies that operate in the fishery in question. This can be done through a broad search of company websites, annual reports, securities filings, or other publicly available information. Alternatively, if the study is focused on a specific fish wholesale company (or companies), then the first step is to identify the fishery or fisheries from which they source their fish.

***Step 2*** is to identify the company’s fleet involved in this fishery. This step is needed if the geographic area of the actual fishing activity is unknown or spread over a large area. For example, tuna fishing companies often target tuna in different oceans or ocean areas, but their fleet can be traced to the tuna RFMOs they participate in, or through vessel monitoring systems (see Step 3 below). A company’s fleet information may be available on their company website, through third-party verification and certification programs, or through available vessel registries.

***Step 3*** is to identify or confirm the fishing area. Some companies report with greater specificity the areas they fish, while others do not. This can be overcome with vessel information if these vessels can be linked to available Automatic Information System or Vessel Monitoring System data (commonly referred to as AIS and VMS, respectively). If this is not possible, the species bio-geographic extent (i.e., species range) would limit the fishing activity or whether the vessels’ flag state has access to or has been observed fishing in certain areas (e.g., other countries’ EEZs).

***Step 4*** is to confirm the fish species and stock being targeted by the company. If a company targets many stocks of the same species, these are treated individually.

***Step 5*** is to gather relevant fisheries information on the stock in question, including biomass, effort and landings data, ex-vessel prices and cost of fishing data. The sources of these data may vary by the stock under study, but include national and regional level data wherever available, and can be supplemented by reconstructed landings [5] and global coverage of the ex-vessel value of fish and cost of fishing [15,16].

***Step 6*** uses published abundance, relative abundance (e.g., catch per unit effort (CPUE)), and catch data as inputs into the Catch-MSY (CMSY) stock assessment method developed to allow evaluation of any fish stock using a Bayesian Schaefer model (BSM) with priors and available catch and biomass information [17,18]. The BSM model uses catch and abundance data to predict values of *r* and *k* based on *a priori* known ranges of the current biomass. This step can also be undertaken with any other stock assessment method, depending on the data-poor or data-rich nature of the stock. However, we chose the highly versatile CMSY method as the default, as it is a method that can easily be applied to all fish stocks around the world, whether previously evaluated or not, and whether the stock and fishery in question is data-rich or data-poor. The version used here has been updated [18] to address earlier challenges including biases in the estimation of stock size and productivity. In addition, the CMSY method in general performed well compared to other stock assessment methods [19]. The results of the CMSY method can be used to model past or future stock biomass scenarios based on different catch strategies to demonstrate the benefits of alternative fishing approaches on future biomass, landings, and revenues. Modeling future scenarios incorporates additional uncertainty, while modeling alternative past scenarios can demonstrate costs and benefits if alternative strategies had been taken and can be used to inform present decisions.

Our study uses the CMSY stock assessment model outputs of MSY as a theoretical benchmark to what these fisheries are measured against. We do not suggest that catches at MSY levels should be constant, as fish stocks are subject to natural variability. In addition, MSY cannot be achieved for all stocks in an ecosystem simultaneously due to predator-prey population dynamics in ecosystems where fishing is occurring [20]. Also, MSY needs to be viewed as a ‘limit reference point’ rather than a ‘target reference point’ for fisheries management [21,22]. Management strategies must take this into account, primarily by aiming for levels of catch below MSY and biomass levels above levels theoretically required to yield MSY (i.e., B_MSY_); or by applying fishing mortality rates (F, estimated by dividing the catch by biomass in a given time) lower than that which would yield MSY (i.e., F < F_MSY_). This ensures that healthy biomass levels can be maintained within functioning marine ecosystems while potentially yielding higher catches at sustainable levels (i.e., provided that current biomass levels are above B_MSY_ and/or that the current yield is below MSY). Through the scenario analysis component of this method, different management strategies can be explored to maintain consistent high biomass (at, or preferably above, B_MSY_), while providing higher fisheries yields. However, fisheries are often required to be managed for MSY including in the United Nations Convention on the Law of the Sea and the European Union’s Common Fisheries Policy [23,24]. Therefore, it is important to test this method for MSY as a target as well as for more precautionary limits including harvest control rules that use a fraction of MSY, commonly termed ‘pretty good yield’ [25].

***Step 7*** uses the above information in conjunction with other company information to evaluate the company’s revenues, and if possible, profits. We estimated a company’s average landings for the most recent five-year period for which information was accessible, based on a company’s ownership of quota, or reported average landings in recent years. We determined a simple estimation of potentially maximum unrealized revenues as determined by the difference between the average current landings and landings at MSY levels, assuming the proportion for each company would remain the same. We expressed unrealized revenues at the company level as related to unrealized ex-vessel value only (i.e., first point of sale, without post-landing processing or value-adding). Finally, we converted unrealized revenues to unrealized profits based on the average cost to catch a tonne of fish in that fishery and subtracting potential costs from potential revenues.

Here, we demonstrate the applicability of this method with two case studies: the Peruvian anchoveta (*Engraulis ringens*; Engraulidae), and the USA’s menhaden fisheries (Atlantic and Gulf menhaden; *Brevoortia tyrannus*; Clupeidae and *B*. *patronus*; Clupeidae, respectively). We also demonstrate a retrospective approach to scenario analysis from Step 6 for the more complex scenario of the anchoveta fisheries. This involved modeling the potential landings, revenues, and profits given alternative fishing mortality rates based on known previous changes. We opted for a retrospective scenario analysis approach here due to the high environmental and biological variability of the anchoveta stock which makes forecasts highly uncertain [26,27]. This scenario analysis can be specified for different outcomes (e.g., higher biomass, more consistent landings, higher revenues), and details on the alternative scenario for the anchoveta fisheries can be found in section 2.2.1. The ex-vessel prices used were adjusted to account for elasticity under higher or lower landings (see S1 Appendix).

### 2.2.1 Peruvian anchoveta case study

The Peruvian anchoveta (*Engraulis ringens*) has two distinct stocks: the North-Central, and the smaller Southern stock, which is shared with Chile [28]. We focused here on the North-Central stock and Peru’s catches from the Southern stock, as Peru contributes most of the world’s anchoveta landings [29]. These fisheries are undertaken solely with purse seines [30], with industrial and semi-industrial vessels off Peru’s coast. In general, these fisheries have low by-catch of other species and no interaction with the seabed. The major challenge for these fisheries is the by-catch of juvenile anchoveta, which negatively affects population growth and hence subsequent landings, and which leads to temporary closures, e.g., of the second 2016 fishing season [31]. These fisheries are reduction fisheries, where virtually all the fisheries landings are ‘reduced’ to fishmeal and fish oil. Peruvian anchoveta was for most of the late 20^th^ century the largest single species fishery in the world with catches exceeding 16 million tonnes for a few years [32,33]. However, the returns of this fishery are highly variable and are influenced strongly by the El Niño and La Niña oceanographic oscillation [27,34,35].

We limited our analysis to the seven largest anchoveta fishing companies in Peru over the 2011–2015 period. We determined vessel ownership through Peru’s *Ministerio de la Producción* (PRODUCE) [30]. As the Peruvian fishery occurs solely within Peru’s EEZ, it was not necessary to confirm vessel-level spatial fishing activity through external sources. We determined company level landings through current fishing quotas allocated to vessels (Table 1), and we assumed all companies captured their quota share of the actual landings in each year. We tested the validity of this assumption against actual proportions of landings over the study period and found no difference in results (see below). We evaluated the catch, abundance data, and biological priors using the CMSY method [18]. Catch and abundance data by season were accessed from Peru’s fisheries management organizations PRODUCE and *Instituto del Mar de Perú* (IMARPE) [36]. The Southern stock, which is shared with Chile, was modeled using reconstructed fisheries catches from Peru and Chile [37,38], and biomass data from FishSource [39] as a relative indicator of abundance. The catches were annually distributed between Peru and Chile based on each country’s known proportional contribution to the total anchoveta catch reported within the stock’s geographical extent. The intrinsic rate of growth parameter (*r*) for use in the CMSY calculations was obtained from FishBase [40] for Peruvian anchoveta using two standard deviations from the mean as the lower and upper bound estimates (1.36–3.17 year^-1^). We evaluated the actual average landings by company from 2011–2015 against the estimated company landings when landings are modeled under the scenario analysis (see below). We used the most recent ex-vessel price available for Peru’s anchoveta fishery of $134 USD·tonne^-1^ based on the landed value of the anchoveta fishery [41]. The cost of fishing was aggregated based on cost and production amount of the two main Peruvian pelagic fleets (steel and wooden purse seiners), which land almost all of their landings for reduction [S1 Table, 27].

**Table 1:** Fishing quota for Peruvian anchoveta and fleet size by company in Peru during 2011–2015.

Company	North-Central stock quota(%)^1^	Southern stock quota (%)^1^	Estimated 2015 landings (10^3^ t)	Estimated 2015 revenue (USD 10^6^)
Tecnologica De Alimentos S.A.	14	17	524	70
Corporacion Pesquera Inca S.A.C.^2^	11	3	368	49
Pesquera Diamante S.A.	9	8	309	41
Austral Group S.A.A	7	4	241	32
CFG Investment S.A.C.^2^	6	11	237	32
Pesquera Exalmar S.A.A.	7	5	236	32
Pesquera Hayduk S.A.	6	3	225	30

^1.^ [30]

^2.^ We present Corporacion Pesquera Inca S.A.C. and CFG Investment S.A.C. separately here, as they were not under the same ownership for the entire study period of 2011–2015.

We used the biological parameters estimated by the BSM and CMSY method (biomass, intrinsic rate of growth (*r*), carrying capacity (*k*), and F_MSY_) to model an alternative scenario over the most recent 15-year period. We then modified the *r* parameter to account for recruitment anomalies, which are common in the Humboldt Current ecosystem [42,43]. This modification was done by minimizing the sum of squared deviations between the CMSY estimated output and the scenario-predicted biomass given the surplus-production function [22,44] by modifying the *r* value each year. This allowed us to estimate biomass and landings under alternative fishing scenarios.

We estimated the target biomass of fish to be excluded from the fishing mortality as is done for the management of the anchoveta stock, hereafter referred to as the biomass reference point [45]. This was done using the F_MSY_ output of the model as a constant. The baseline scenario was defined as the observed biomass and catches from 2000–2015. The first alternative scenario (hereafter referred to as the Optimized F scenario) was to optimize fishing mortality (F; restricted to be below F_MSY_ model output) to achieve a higher or equal biomass one year after the time series than present, higher average biomass than under the baseline scenario, and to maximize the difference between the landings over the time period. F_MSY_ was set as an upper limit as this ensures a more conservative approach where F_MSY_ can only be achieved in years with biomass equal to or above B_MSY_. All optimization scenarios were estimated using Microsoft Excel’s Solver function using the generalized reduced gradient nonlinear solution method. Once the alternative scenario was complete, we re-modeled with the current policy to apply fishing mortality obeying a biological reference point, and without this restriction. The second alternative scenario (hereafter defined as (‘pretty good yield’ [PGY]) applied an alternative precautionary harvest control rule of catching MSY·0.91 when B>B_MSY_ and linearly adjusting catches downwards until B = 0.5 BMSY where catches are reduced to zero [46].

### 2.2.2 Menhaden case study

Another major, although much smaller, reduction fishery is for Atlantic (*Brevoortia tyrannus*) and Gulf menhaden (*B*. *patronus*) in the Southeastern USA. There, menhaden play an important role in ecosystems as a forage fish species, i.e., a major source of food for higher-trophic level organisms [47,48]. The fisheries for these two species are managed at a regional level by the Atlantic and Gulf States Marine Fisheries Commissions (ASMFC and GSMFC, respectively).

Omega Protein (owned by Cooke Aquaculture) takes over 75% of all Atlantic menhaden landings [49] and jointly, Omega Protein Corporation and Daybrook Fisheries (owned by the Oceana Group) are the major fishing companies for Gulf menhaden. These companies operate in federal- and state-controlled waters of the USA and are thus subject to the regulations of the ASFMC and GSMFC. These two species are fished exclusively with purse seines when caught by reduction fisheries [50], and have very low rates of by-catch and discards [51]. If considered together, the menhaden fisheries are the second largest fishery in US waters by tonnage [50], even though they are much smaller than in the past [52].

We determined company vessel ownership through company reports [53] and third-party sources [54,55]. Landings data were obtained from the National Marine Fisheries Service using taxon identifier ‘Menhaden’ separated by Atlantic and Gulf regions [50]. Minor landings of other menhaden species (i.e., *B*. *gunteri* and *B*. *smithi*) may be included in the data from the National Marine Fisheries Service, but are unlikely to influence the general pattern observed. Catch per unit effort data were used as an indicator of relative abundance. CPUE data were obtained from each stock’s most recent stock assessment [47,48]. The intrinsic rate of growth parameter (*r*) was obtained from FishBase [40] using two standard deviations from the mean as the lower and upper bound estimates for Atlantic and Gulf menhaden (0.43–1.16 and 0.32–1.38 year^-1^, respectively). The landings, CPUE, and biological priors were used in the BSM and CMSY method [18]. The most recent ex-vessel prices were used from the National Marine Fisheries Service [50]. The cost of fishing was based on Atlantic menhaden, taking total costs of their operations minus processing plant labor costs (S1 Table) [56]. While this may slightly overestimate the cost per tonne of catch, it kept our estimates of potential profits conservative and thus this estimation was used for both Atlantic and Gulf menhaden.

As there are only two companies that fish Gulf menhaden, their actual individual landings are obscured by privacy laws [57]. However, separating their catch by known catch proportions reported by each company was possible based on landings by area and company reports [49,53,58].

## 3. Results

### 3.1 Peruvian anchoveta

**Fig 2:**
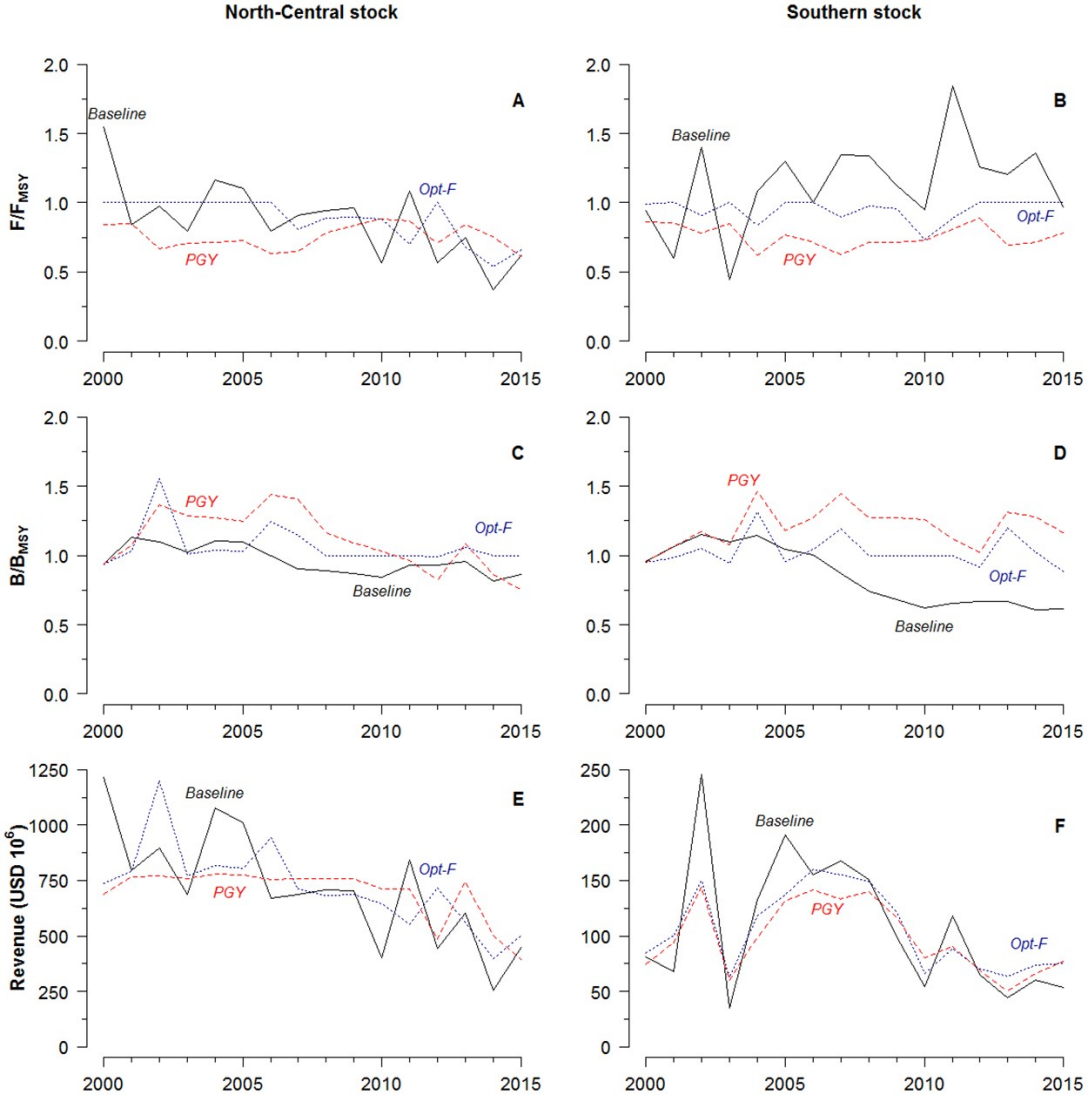
Fishing mortality (F/F_MSY_), biomass (B/B_MSY_), and fisheries revenues for the North-Central and Southern Peruvian anchoveta stocks under baseline, Optimized F (Opt-F) and pretty good yield (PGY) scenarios.

We estimated that the current biomass levels of Peruvian anchoveta -of both stocks- were below that which would potentially yield MSY (Fig 2), suggesting that the stocks are in a depleted state (B < B_MSY_). However, recent levels of fishing mortality rates are below the fishing mortality at MSY (F < F_MSY_), signaling that overfishing was not occurring (Fig 2).

While the anchoveta stocks are naturally volatile in their biomass (due to sensitivity to highly variable oceanographic conditions), ensuring the stocks remain at higher biomass levels (i.e., biomass levels that could theoretically provide MSY) could have increased yields for the most recent period (Fig 2, Table 2, and S2 Table). Average annual landings over the 2011–2015 period modelled here under the hypothetical, optimized-F scenario could have increased from 3.88 million tonnes to 4.16 million tonnes (± 940,000 tonnes) for the North-Central stock, and from 509,000 tonnes to 563,000 tonnes (± 58,000 tonnes) for the Southern stock. Overall, the average landings over the 2011–2015 period could have been increased by about 330,000 t·year^-1^. In this scenario, the North-Central stock would be responsible for 60% of the total potential increase in landings, although its productivity would only increase by 7%. The application of the optimized-F fishing strategy on the shared stock could increase total landings by 47% in 2015.

**Table 2:** Average biomass and landings outputs (10^3^ t) of the scenario analysis for North-Central and Southern anchoveta stocks of Peru.

Scenario (Biomass)^2^	North-Central		North-Central		North-Central		South^1^		South^1^		South^1^	
	Baseline		Optimized-F (807)^2^		PGY (807)^2^		Baseline		Optimized-F (0)^2^		PGY	
Years	Biomass	Landings	Biomass	Landings	Biomass	Landings	Biomass	Landings	Biomass	Landings	Biomass	Landings
2000–2005	10,319	7,093	10,654	6,380	11,597	5,589	3,133	940	3,009	817	3,351	749
2006–2010	8,742	4,744	10,444	5,602	11,883	5,713	2,281	939	3,050	981	3,800	916
2011–2015	8,711	3,880	9,781	4,158	8,709	4,330	1,873	509	2,926	563	3,440	532
Mean (10^3^ t)	9,324	5,355	10,315	5,443	10,784	5,234	2,473	805	2,996	789	3,519	733
Coefficient of Variation (%)	10.5	36.0	13.9	26.3	19.0	16.9	24.8	57.0	11.0	33.3	11.7	31.2
95% CI (10^3^ t)	482	946	705	702	1,003	433	301	225	162	129	202	112

^1.^ The Southern stock biomass refers to the whole stock which is shared with Chile, but the landings reflect only Peru’s landings of the Southern stock as this is the focus of this study.

^2.^ The number in brackets refers to the biomass (10^3^ t) reference point, i.e. the biomass not subject to fishing mortality each year for the scenarios as established based on the scenario analysis methods

In reality, landings were lower due to the stocks being in depleted states, which even prompted the shortening of several fishing seasons in recent years (S2 Table). The landings under the Optimized-F scenario oscillate between being higher and lower than the realized landings in each year, but on average are higher for the North-Central stock, and could have been achieved with a higher and more stable biomass than observed in reality for both stocks (Table 2).

The benefits highlighted above in the most recent period are driven by reducing fishing mortality to at or below F_MSY_ over the period of 2000–2010 leading to higher and more stable biomass. This can be achieved while keeping average landings over these 11 years nearly identical, although in most years the catches would have to be lower than they were under the baseline scenario (S2 Table). With a more conservative harvest control rule (PGY), the average landings in the first 6 years are much lower than the baseline (5,589 compared to 7,093 thousand tonnes), but are also higher for both of the following 5 year periods in the North-Central stock (Table 2). These lower catches for the first 10 years would translate into lower fisheries revenues for the companies targeting these stocks, although the result under either of the alternative strategies (F_MSY_ or PGY) leads to higher and more consistent biomass and landings.

The seven largest companies involved in the anchoveta fishery in Peru control about 60% of the quota for the North-Central stock, and about 50% of the Peruvian share of the Southern stock (Table 1). The average unrealized potential revenues per company (depending on their quota share) ranged between USD 3.0 million and USD 9.1 million (Table 3). When accounting for the additional cost of fishing if catches could have been taken to MSY, the total unrealized potential profit would have been approximately USD 8 million. We compared the results whether we use current quota percentage (S3 Table) or historical landings proportions to assign landings to companies, and the difference in results were minor (S4 Table).

**Table 3:** Average attained landings for the top seven companies for 2011–2015 (10^3^ t; accounting for 57% of landings in Peru), scenario landings (10^3^ t) and their impact on revenue and profits (USD 10^6^) for F_MSY_ and PGY scenarios.

Scenario	Company	Landings^1^	Scenario landings^2^	Unrealized revenue	Cost of landings shortfall	Unrealized profits
F_MSY_ Scenario	Tecnologica de Alimentos S.A.	616	685	9.14	6.93	2.20
	Corporacion Pesquera Inca S.A.C.	429	464	4.64	3.51	1.13
	Pesquera Diamante S.A.	363	401	5.07	3.84	1.22
	Austral Group S.A.A.	282	307	3.41	2.58	0.83
	Pesquera Exalmar S.A.A.	277	303	3.49	2.64	0.84
	Pesquera Hayduk S.A.	262	285	3.01	2.28	0.73
	CFG Investment S.A.C.	280	317	4.87	3.70	1.17
PGY Scenario	Tecnologica de Alimentos S.A.	616	704	11.64	8.85	2.78
	Corporacion Pesquera Inca S.A.C.	429	481	6.94	5.27	1.67
	Pesquera Diamante S.A.	363	413	6.64	5.05	1.59
	Austral Group S.A.A.	282	318	4.80	3.65	1.15
	Pesquera Exalmar S.A.A.	277	313	4.81	3.66	1.15
	Pesquera Hayduk S.A.	262	295	4.36	3.32	1.05
	CFG Investment S.A.C.	280	324	5.78	4.40	1.38

^1.^ See S2 Table for estimated company landings

^2.^ Optimized-F landings are generated by optimizing the biomass reference point, and fishing at F_MSY_ when B ≥_B_MSY_

Another outcome of the scenario analysis was the potential decrease in inter-annual variability under the F_MSY_ strategy. Under the Optimized-F scenario (i.e., fishing at F_MSY_ when B ≥ B_MSY_), the coefficient of variation (a measure of the relative variability, calculated as the standard deviation divided by the mean times 100) for potential landings decreases for both stocks, suggesting reduced inter-annual variability. While the potential increase in landings was modest, the biomass of both stocks would be at higher average levels and the variability in landings would likely be lower or similar for the Northern stock, thus implying more consistent landings and revenue for fishing companies.

The costs and benefits of rebuilding the Southern stock are not equally shared by Peru and Chile. Optimized-F Chilean anchoveta landings corresponding to the shared stock increased in both periods (2000–2010, and 2011–2015) and at much higher rates than they did in Peru (Table 4). The losses from rebuilding during the first ten years are not compensated for over the whole period although the most recent landings are higher on average.

**Table 4:** Peru and Chile’s average actual and modeled landings (10^3^ t) of the shared Southern Peru/Northern Chile stock over two time periods.

	Actual		Optimized-F		PGY	
Years	Peru	Chile	Peru	Chile	Peru	Chile
2000–2010	940	790	892	825	825	772
2011–2015	509	1,006	563	1,177	532	1,079

### 3.2 Menhaden

The most recent landings included in this study (2012–2016) for Atlantic and Gulf menhaden were at levels below the estimated MSY (Table 5 and S4 Table). In addition, the biomass of each stock was estimated as being above B_MSY_ (B > B_MSY_) and the fishing mortality was below F_MSY_ (F < F_MSY_) according to our model results (Table 5). Therefore, the stocks appear neither overfished nor being overfished.

**Table 5:** Realized landings (10^3^ t), MSY (10^3^ t), and catch-based indicators of stock status for Atlantic and Gulf menhaden.

Menhaden stock	2015 Landings^1^	MSY^2^	B/B_MSY_^2^	F/F_MSY_^2^
Atlantic	201	340	1.38	0.278
Gulf	539	698	1.18	0.500

^1.^[50]

^2.^CMSY model output

Omega Protein and Daybrook Fisheries account for the bulk of all menhaden landings in the USA (Table 6). The total revenue of these two fisheries was approximately USD 140 million annually. However, landings were below MSY, and if increased to the theoretical MSY level could potentially increase economic returns from the fishery (Table 7). The cumulative unrealized potential revenue (USD 72 million) for these two companies (Table 7) was around 50% of the current total fisheries revenues (USD 140 million; Table 6). In addition, when accounting for potential fishing costs for these unrealized landings as well as the lower ex-vessel price given the elasticity (see S1 Appendix), there were unrealized potential profits of around USD 15 million. The findings were similar for the PGY scenario although somewhat lower due to the lower yields when the stock is in a healthy state as it is at present. Even with these precautionary limits, the fishery could have additional revenues of over 50 USD annually and profits of around 12 million USD annually.

**Table 6:** Major menhaden fishing companies in the United States of America.

Company	Menhaden stock	Landings (%)	Estimated 2016 landings (10^3^ t)	Estimated 2016 revenue ($ 10^6^)	Source
Daybrook Fisheries	Gulf	~40.0	194	50	[53], [58]
Omega Protein	Gulf	48.5	235	60	[49]
Omega Protein	Atlantic	76.8	144	29	[49]

**Table 7:** Average landings (10^3^ t), potential MSY (10^3^ t), and associated unrealized revenues (USD 10^6^) for menhaden by the major fishing companies in the USA.

Scenario	Company	Menhaden	Mean landings^1^ (2012–2016)	Potential landings	Unrealized revenue	Fishing cost at MSY	Unrealized profits
MSY	Daybrook Fisheries	Gulf	188	279	22.58	16.20	6.38
	Omega Protein	Gulf	228	338	27.37	19.64	7.74
	Omega Protein	Atlantic	147	262	22.09	20.39	1.70
PGY	Daybrook Fisheries	Gulf	188	254	16.36	11.74	4.62
	Omega Protein	Gulf	228	308	19.83	14.23	5.61
	Omega Protein	Atlantic	147	238	17.56	16.20	1.36

^1.^ See S2 Table for estimated company landings

## 4. Discussion

### 4.1 Overall

Here, we were able to apply this method to two large case studies and the nine major companies involved in their exploitation. The total potentially unrealized annual profits for the two case studies presented here are USD 8 million for the Peruvian anchoveta and nearly USD 16 million for the US menhaden fisheries. These unrealized profits are based on the assumption that these fisheries can be managed to maintain biomass levels equal to or larger than required to produce MSY (B_MSY_) on an ongoing basis, and fisheries yield can be at MSY or F_MSY_ when biomass is below B_MSY_. For clarity and simplicity, we demonstrated these potential unrealized benefits for two low-value fisheries, with high landings, and heavily concentrated fishing company actors. The biomass of the anchoveta stocks are below levels that optimize or maximize potential catches (i.e., B_MSY_ levels), and thus would benefit from stock rebuilding. In contrast, the menhaden stocks are at healthy biomass levels with regards to estimated MSY, and there is thus flexibility for carefully increased catch, revenue and profits. These unrealized revenues and profits can motivate stakeholders at different levels in the fisheries sector, including investors in these companies, due to the unrealized potential benefits originating from suboptimal levels of fisheries stocks. In addition, fisheries with healthy biomass levels are more likely to qualify for eco-certification programs [59] that may receive a price premium [60] giving an extra incentive for these companies.

While the present study has focused our approach on fisheries examples where the stock biomass is lower (anchoveta) or higher than B_MSY_ (menhadens), there is also the extreme case of overfishing leading to collapsed stocks. While not fitting the strict economic definition of ‘stranded assets’, where assets must be subject to regulatory or legislative changes rather than biological changes [61], fish stocks that collapse are indeed a form of ‘lost’ or unusable assets. For companies invested in such heavily overfished or even collapsed stocks that are then likely subject to more stringent limitations of fishing pressure, these fish stocks represent a form of stranded assets whose values to the companies are reduced [61]. When quotas for stocks cannot be realized due to low biomass of the fish stock, the owner’s resource rents are reduced to zero. However, even without fishing and resource rents being reduced to zero, continued fishing of stocks in a suboptimal state reduces long-term revenues. Thus, the concept of stranded assets can be extended to where overexploitation reduces the value of fisheries assets marginally or completely.

The scenario analysis of the anchoveta fisheries conducted here, comparing the Optimized-F scenario and the PGY scenario to the baseline scenario of actual fisheries and stock conditions illustrated differences in landings and revenues under different management scenarios. While we were able to demonstrate potentially more optimal fishing mortality rates to maximize these differences in the Optimize-F scenario, it is somewhat unrealistic for fisheries managers as they operate in an environment of imperfect information. However, the PGY scenario often had similar results with clear rules that can be operated on given knowledge about the status of the stock. The catch and revenue difference can be substantial over the time period analyzed, but on an annual basis are quite comparable for the Northern stock. Thus, it demonstrates the current high performance of the managers to maximize anchoveta yields and revenues of this fishery even though they could be higher if B > B_MSY_ as in the case of the menhaden fisheries.

We compared current and past landings to landings under modeled scenarios that rebuild the stocks with either optimal fishing mortality rates or a precautionary harvest control rule (0.91·MSY). It is important to recognize that multiple fisheries on a variety of species in the same area cannot achieve MSY simultaneously for all species due to ecosystem interactions [20]. It is thus important to retain a precautionary approach and not maximize landings (in terms of MSY) and ignore the ecological function that species fill in their ecosystem [62]. The PGY scenario models this more closely to reality as there is a precautionary limit placed on MSY with reductions for when the stock is below B_MSY_. Our example for anchoveta would eventually lead to increased landings and revenues, but only after having increased biomass and decreased annual variability in biomass. Our case study for Gulf menhaden was informed by the GSFMC that incorporates menhaden’s role as a forage fish into their fishing limits [48].

Both case studies demonstrated the benefits of rebuilding fisheries stocks at the scale of individual companies acting in these fisheries. In the case of the anchoveta, currently unrealized benefits could only be attained after rebuilding the stock biomass to levels above B_MSY_, while the menhaden fishery with healthy biomass levels illustrated the ready availability for increased landings and profit potential, should appropriate economic drivers emerge such as increased prices for fishmeal or lower costs of fishing. It may be helpful to quantify potentially unrealized benefits at different scales and to express them in units relevant to the different stakeholders in the fisheries sector and beyond. Not only could it be important to those actors directly involved, such as company owners, shareholders and investors, but also to other important stakeholders such as governments that face foregone tax revenue or licensing fees due to overfished stocks or by not optimizing potential benefits of their natural resources. Unfortunately, governments far too often support fisheries well past the point of being economically profitable within ecologically sustainable limits through extensive harmful subsidies [63,64].

### 4.2 Anchoveta

The anchoveta stocks are still in a depleted state. This is partially due to overfishing, particularly in the Southern stock, but also due to fluctuations driven by El Niño and La Niña events, and increasing variability due to climate change [34,65]. Our findings generally concur with the latest numbers of the national stock assessments conducted by PRODUCE and Chile’s Instituto de Fomento Pesquero [36,39]. The large differences in the modeled scenarios’ landings for the Southern stock are due to the current low biomass of leading to lower catches over the earlier years, whereas, the North-Central stock is fished close to the level in the Optimized-F and PGY scenarios proposed here. The North-Central stock’s biomass declined recently in the observed and modeled scenarios due to strong recruitment anomalies caused by recent El Niño and La Niña events [39,66]. Both anchoveta stocks could have delivered higher landings in the past if biomass levels would have been maintained at higher levels by pro-active management action. Increasing the anchoveta biomass by reducing fishing mortality rates to levels at or below F_MSY_ or instituting a precautionary limit on MSY (see Methods for further details) could benefit these fishing companies in the future.

However, it is important to highlight that rebuilding the stocks has a cost. The proposed theoretical MSY strategy generated losses in the earlier years of its implementation, particularly in the Southern stock (Fig 2). This shows that rebuilding the shared stock has an initial cost for Peruvian companies, but they benefit from the increased (and likely more stable) landings that result from a stock with higher average biomass in the later period (i.e., in the medium-term: after 10 years). Reported landings in Peru were higher than the scenarios’ landings for those initial years because the model restricted the higher levels of fishing to prevent overfishing the stock (F > F_MSY_). Thus, the current low landings reported by Peru in the Southern stock are likely due to continued overfishing by both countries pressuring the stock into a depleted state (Fig 2).

Recent landings of anchoveta have been volatile (S2 and S3 Tables), although they were much more variable historically, particularly in the 1970s and 1980s. The current management regime based on individual transferrable quotas (ITQ) has the potential to improve the state of the anchoveta resource and fisheries over time [67], as long as the high data and assessment requirements for ITQ systems are maintained and management action is swift and proactive with regards to setting and adjusting annual allowable catch limits [68]. However, environmental fluctuations and the large effect of El Niño and La Niña events may continue to have negative repercussions for this industry [34]. Based on current fishing exploitation [36,69], there could be greater benefits realized in terms of higher landings and higher economic returns if biomass levels were to be increased and maintained at higher levels based on our estimates of B_MSY_. A constant MSY does not optimize yields of this fishery due to the high variation in stock sizes [70], but policies that optimize and adjust fishing mortality rates annually based on overall higher biomass levels have the potential to achieve higher landings and thus revenues.

It is particularly important to highlight that the unrealized potential benefits of the industrial anchoveta fishery could be much higher if alternative uses for the landings were considered, namely direct human consumption rather than reduction [30,35]. Based on local value-chain multipliers [30] and the reported landings of anchoveta in 2013, a transition from the current use scheme (reduction) to a scenario were all landings were used by the canning industry for direct human consumption would result in up to 20% increases in net profits, a near doubling of employment, and an additional 700,000 tonnes a year of seafood for the human consumption market, while decreasing fishmeal production by half [35]. However, this reduction of fishmeal production from one of the largest supplies would obviously have negative effects for fishmeal intensive forms of aquaculture.

The Southern stock of anchoveta is expected to shift its distribution further south due to climate change where the catches will no longer be available in Peru’s EEZ [71]. This means the share of this stock available to Peruvian companies will likely decline in the long-term, but our results show that these companies can benefit in the short to medium term (5–15+ years) from reducing fishing pressure and rebuilding the stock. The current overfished state of the stock is having negative repercussions for fishing companies in Peru and Chile, and the stocks being rebuilt could deliver higher landings and revenues in the future. While Chile is expected to have larger fisheries landings and revenues under the modeled scenario and into the future, this does not preclude Peru from cooperating in an agreement to rebuild the Southern stock. The short-term costs borne by Peru combined with the majority of benefits being derived by Chile could explain why Peruvian companies have been reluctant to support joint management of the shared stock. Finally, although not considered here, the bulk (~90%) of Chile’s landings of the shared stock is caught by a single company that could benefit considerably from the rebuilding strategies modeled here.

### 4.3 Menhaden

The CMSY stock assessment method used here was in agreement with other published assessments on these stocks that biomass levels are above those potentially supporting MSY level catches and fishing mortalities are below MSY levels [47,48]. These results of potentially higher landings for MSY must be qualified as they are based on a single stock and do not consider the important ecosystem implications of menhaden [47,48]. Thus, current fisheries pressures on these stocks should be considered more precautionary and in-line with ecosystem considerations in fisheries than any attempts to maximize landings and economic returns via fishing at MSY.

For Gulf menhaden, the responsible fisheries management agency (Gulf States Marine Fisheries Commission, GSMFC) increased the catch target reference point by over 70% to 829,737 t·year^-1^ [72], even after accounting for the important ecosystem role of menhaden under ecosystem-based fisheries management [48]. However, only around 436,000 t are expected to be caught [73]. There are four likely explanations for the continuing lower actual fishing levels: i) the demand for menhaden fishmeal is not high enough to encourage heavier fishing; ii) the increases in fishing costs past a certain level of catch makes it less profitable to continue fishing, iii) the price negatively responds to an increased supply, which makes it more profitable to stop fishing early, or iv) a conservative rate of exploitation given broader ecosystem concerns. We demonstrated that increased supply does cause the ex-vessel price to decrease, but it was highly inelastic (see S1 Appendix). If the total of the catch reference point proposed by the management agency were to be realized, Omega Protein’s theoretical unrealized maximized proportion could reach over 400,000 t·year^-1^, and Daybrook Fisheries’ over 330,000 t·year^-1^. These increased catches are above our estimates generated by the CMSY method (Table 7), and the difference in catches is attributed to a lower estimate of MSY from our model that could be driven by differences in input parameters.

While our results suggest that landings could be increased to achieve higher yields (i.e., to theoretical MSY levels), this may negatively affect profitability because of increased fishing costs. In addition, the importance of precautionary ecosystem-based management should limit future catches of menhaden in both ecosystems to ensure enough biomass remains for higher level predators. This is especially important if the current baseline data being used is not truly reflective of long-term historical baselines, which have largely been forgotten [74]. In addition, there are other important fisheries in the ecosystem, and there is evidence that previous fishing of menhaden has negatively impacted other species and their fisheries, including the striped bass recreational fishery [75].

### 4.4 Limitations

A limitation of the research presented here is that it does not consider the trade-offs these companies may be making with higher levels of fishing. A higher level of biomass in the ecosystem does have unambiguous positive effects. Although the three species and four stocks included in this study are all forage fish and thus represent an important part of their respective ecosystems as major food item for higher predators, these predators can also increase or decrease the populations of their prey due to their own changes in abundance. Alternatively, higher levels of fishing on these forage fish may reduce populations of other species, which may negatively influence fisheries or the populations of non-targeted species. Such broader ecosystem effects need to be considered carefully and precautionary in ecosystem-based management of fisheries.

Some of the data needed for this study were not readily available or are subject to continually changing conditions. This study represents a snapshot in time and extrapolated the values at present (such as percentage of quota held by a company’s vessels) to try and understand how changing conditions could affect these companies. We sought to minimize parameter and structural uncertainties in our method by relying on the best available data from a diversity of sources. However, some assumptions had to be made to distribute landings (such as company reported landings for menhaden in the Gulf and Atlantic fisheries) and thus revenues to companies. We used a conservative price-elasticity estimated for the menhaden fisheries to qualify future revenues subject to potential increases in landings. Regardless, the examples presented here are a first application of a method that can continue to be improved upon with additional estimates and data.

Future research should focus on applying this method to a wider range of fisheries and companies, including severely data limited cases, as may be the case in developing countries. The ability to perform this analysis in data limited cases will vary, but the several public and global databases described in the methods should allow at least a coarse idea of the benefits and costs of rebuilding fisheries. In addition, it would be valuable to include additional dimensions to improve the financial analysis, including price-effects of increased supply and relevant costs of fishing at different fishing effort levels [76]. Also, it is important to consider if there are other costs associated with reductions in fisheries effort and catches [2] and how these may affect individual fisheries. Finally, alternative uses, from canning for direct human consumption to Omega-3 production for the pharmaceutical industry, as well as the importance of the ecological role played by the target species (e.g., forage fish, predator), should also be considered when assessing the potential unrealized benefits of a fishery at company level, as companies might have interests in multiple end-uses and target various species within the same ecosystem.

## 5. Conclusion

We present a method for relating a company’s fisheries quotas and landings from the stocks they exploit to potential unrealized revenues and profits due to sub-optimal stock biomass and landings. We believe this approach can be used both at an aggregate level and at a micro level to analyze the state of fisheries stocks and the effects on balance sheets of fishing companies. This method imposes traceability on companies by piecing together these disparate parts, rather than wait for voluntary measures to be adopted. This is an important step to be taken to advance transparency in seafood supply chains.

## Supporting information

S1 Table Cost of fishing and ex-vessel prices by fishery (USD·tonne^-1^). (DOCX)

S2 Table Scenario analysis for north-central and southern anchoveta stocks of Peru. (DOCX)

S3 Table Landings (10^3^ t) by company by year for the Peruvian anchoveta fishery based on quota ownership. (DOCX)

S4 Table Average attained landings for 2011–2015 (10^3^ t), modeled higher landings (10^3^ t) based on Optimal F, and their impact on revenue and profits (USD 10^6^). (DOCX)

S5 Table Estimated landings (10^3^ t) by company for the US menhaden fishery. (DOCX)

S1 Appendix Supplemental methods. (DOCX)

S1 File Supplementary data. (XLSX)

## References

[1] World Bank. The Sunken Billions Revisited: Progress and Challenges in Global Marine Fisheries. Washington, DC; 2017.

[2] Sumaila UR, Cheung W, Dyck A, Gueye K, Huang L, Lam V, et al Benefits of rebuilding global marine fisheries outweigh costs. PLoS One. 2012;7 10.1371/journal.pone.0040542 22808187 10.1371/journal.pone.0040542 PMC3396648

[3] Costello C, Kinlan BP, Lester SE, Gaines SD. The Economic Value of Rebuilding Fisheries. OECD Food, Agric Fish Pap. 2012; 1–68. 10.1787/5k9bfqnmptd2-en OECD

[4] Costello C, Ovando D, Hilborn R, Gaines SD, Deschenes O, Lester SE. Status and Solutions for the World’s Unassessed Fisheries. Science (80-). 2008;338: 517–520. 10.1126/science.1223389 23019613

[5] Pauly D, Zeller D. Catch reconstructions reveal that global marine fisheries catches are higher than reported and declining. Nat Commun. Nature Publishing Group; 2016;7: 1–9. 10.1038/ncomms10244 26784963 10.1038/ncomms10244 PMC4735634

[6] Asche F, Bellemare MF, Roheim C, Smith MD, Tveteras S. Fair Enough? Food Security and the International Trade of Seafood. WORLD Dev. 2015;67: 151–160. 10.1016/j.worlddev.2014.10.013

[7] Agnew DJ, Pearce J, Pramod G, Peatman T, Watson R, John R, et al Estimating the Worldwide Extent of Illegal Fishing. PLoS One. 2009;4 10.1371/journal.pone.0004570 19240812 10.1371/journal.pone.0004570 PMC2646833

[8] Jaquet J, Pauly D. Trade secrets: Renaming and mislabeling of seafood. Mar Policy. Pergamon; 2008;32: 309–318. 10.1016/J.MARPOL.2007.06.007

[9] Marine Stewardship Council. Teeming with life: A summary of the Marine Stewardship Council’s Strategic Plan, 2017–2020. 2017.

[10] SeafoodSource. 50 NGOs critique MSC over bycatch. In: SeafoodSource [Internet]. 2017 [cited 11 Apr 2018]. https://www.seafoodsource.com/news/environment-sustainability/50-ngos-critique-msc-over-bycatch

[11] Jacquet J, Pauly D, Ainley D, Holt S, Dayton P, Jackson J. Seafood stewardship in crisis. Nat 2010 467 7311. Nature Publishing Group; 2010; 10.1038/467028a 20811437

[12] MAREX. “Sustainable” Shrimp Trawler Arrested in Liberia. In: Maritime Executive [Internet]. 2017 [cited 3 Jun 2018]. https://www.maritime-executive.com/article/sustainable-shrimp-trawler-arrested-in-liberia

[13] Österblom H, Jouffray J-B, Folke C, Crona B, Troell M, Merrie A, et al Transnational Corporations as ‘Keystone Actors’ in Marine Ecosystems. PLoS One. 2015;10: e0127533 10.1371/journal.pone.0127533 26017777 10.1371/journal.pone.0127533 PMC4446349

[14] Aurelius Value. Pingtan Marine: A Fraud That Finances Human Trafficking and Poaching—Aurelius Value. In: Aurelius Value [Internet]. 2017 [cited 5 Jun 2018]. http://www.aureliusvalue.com/research/pingtan-marine-fraud-finances-human-trafficking-poaching/

[15] Tai TC, Cashion T, Lam VWY, Swartz W, Sumaila UR. Ex-vessel fish price database: disaggregating prices for low-priced species from reduction fisheries. Front Mar Sci. 2017;4: 1–10.

[16] Sumaila UR, Marsden AD, Watson R, Pauly D. A global ex-vessel fish price database: Construction and applications. J Bioeconomics. 2007;9: 39–51. 10.1007/s10818-007-9015-4

[17] Martell S, Froese R. A simple method for estimating MSY from catch and resilience. Fish Fish. 2013; 504–514. 10.1111/j.1467-2979.2012.00485.x

[18] Froese R, Demirel N, Coro G, Kleisner KM, Winker H. Estimating fisheries reference points from catch and resilience. Fish Fish. 2017;18: 506–526. 10.1111/faf.12190

[19] Rosenberg AA, Fogarty MJ, Cooper AB, Dickey-Collas M, Fulton EA, Gutierrez NL, et al Developing new approaches to global stock status assessment and fishery production potential of the seas. FAO Fisheries and Aquaculture Circular. Rome; 2014.

[20] Walters CJ, Christensen V, Martell SJ, Kitchell JF. Possible ecosystem impacts of applying MSY policies from single-species assessment. ICES J Mar Sci. 2005;62: 558–568. 10.1016/j.icesjms.2004.12.005

[21] Caddy JF, Mahon R. Reference points for fisheries management. FAO Fish Tech Pap. 1995;341: 1–83.

[22] Hilborn R, Walters CJ. Quantitative fisheries stock assessment: choice, dynamics and uncertainty [Internet]. New York: Chapman & Hall; 1992 https://books.google.ca/books?id=Y0EGCAAAQBAJ&dq=Walters+Hilborn&lr=&source=gbs_navlinks_s

[23] United Nations. United Nations convention on the law of the sea [Internet]. UN 1982. 10.1163/15718089720491594

[24] Regulation (EU) No 1380/2013 of the European Parliament and of the Council of 11 December 2013 on the Common Fisheries Policy, amending Council Regulations (EC) No 1954/2003 and (EC) No 1224/2009 and repealing Council Regulations (EC) No 2371/2002 and (EC) No 639/2004 and Council Decision 2004/585/EC. https://eur-lex.europa.eu/LexUriServ/LexUriServ.do?uri=OJ:L:2013:354:0022:0061:EN:PDF

[25] Hilborn R. Pretty Good Yield and exploited fishes. Mar Policy. Pergamon; 2010;34: 193–196. 10.1016/J.MARPOL.2009.04.013

[26] Brochier E, Echevin V, Tam J, Chaigneau A, Brochier T, Echevin V, et al Climate change scenarios experiments predict a future reduction in small pelagic fish recruitment in the Humboldt Current system. Glob Chang Biol. 2013;19: 1841–53. 10.1111/gcb.12184 23554213 10.1111/gcb.12184

[27] Espinoza-Morriberón D, Echevin V, Colas F, Tam J, Ledesma J, Vásquez L, et al Impacts of El Niño events on the Peruvian upwelling system productivity. J Geophys Res Ocean. 2017;122: 5423–5444. 10.1002/2016JC012439

[28] Mendo J. Electrophoretic Studies of Peruvian Anchoveta Engraulis ringens Confirm the Existence of Distinct North/Central and Southern Stocks In: Pauly D, Ruiz-Leotaud V, editors. Marine and Freshwater Miscellanea Fisheries Centre Research Reports. Vancouver: Institute for the Oceans and Fisheries, University of British Columbia; 2018 pp. 16–26.

[29] FAO. Fishery Statistical Collections: Global capture production. (1950–2015). Accessed through FishStatJ software. Rome; 2017.

[30] PRODUCE. Embarcaciones Pesqueras [Internet]. 2017 [cited 27 Jul 2017]. http://www.produce.gob.pe/index.php/shortcode/servicios-pesca/embarcaciones-pesqueras

[31] FAO. High juvenile presence in Peru ends an already tough year for fishmeal production. In: GLOBEFISH [Internet]. 2017 [cited 13 Oct 2017]. http://www.fao.org/in-action/globefish/market-reports/resource-detail/en/c/896543/

[32] Castillo S, Mendo J. Estimation of unregistered Peruvian anchoveta (Engraulis ringens) in official catch statistics, 1951 to 1982 In: Pauly D, Tsukayama I, editors. The Peruvian Anchoveta and Its Upwelling Ecosystem: Three Decades of Change. Manilla, Philippines: ICLARM studies and reviews; 1987 pp. 109–116.

[33] Mendo J, Wosnitza-Mendo C. Peru In: Pauly D, Zeller D, editors. Global atlas of marine fisheries: a critical appraisal of catches and ecosystem impacts. Washington, DC: Island Press; 2016 p. 366.

[34] Ñiquen M, Bouchon M. Impact of El Niño events on pelagic fisheries in Peruvian waters. Deep Res Part II Top Stud Oceanogr. 2004;51: 563–574. 10.1016/j.dsr2.2004.03.001

[35] Bertrand S, Dewitte B, Tam J, Díaz E, Bertrand A. Impacts of Kelvin wave forcing in the Peru Humboldt Current system: Scenarios of spatial reorganizations from physics to fishers. Prog Oceanogr. Elsevier Ltd; 2008;79: 278–289. 10.1016/j.pocean.2008.10.017

[36] PRODUCE. Evaluación de las cuotas individuales transferibles en la pesquería de anchoveta peruana (Engraulis ringens) Stock Norte-Centro [Internet]. Lima, Peru; 2016. http://www.produce.gob.pe/index.php/dgchi/publicaciones

[37] Mendo J, Wosnitza-Mendo C. Reconstruction of total marine fisheries catches for Peru: 1950–2010. Vancouver, BC; 2014. Report No.: 21.

[38] Van Der Meer L, Arancibia H, Zylich K, Zeller D. Reconstruction of total marine fisheries catches for mainland Chile (1950–2010) [Internet]. Vancouver; 2015. https://sau-technical-reports.s3.amazonaws.com/152_van%20der%20meer%20et%20al_2015_Chile%20Mainland_WP.pdf?Signature=%2FuRpSXDaVFs%2F2drLDzZv%2BSMk8Qo%3D&Expires=1524846210&AWSAccessKeyId=ASIAIAD6BN6URY2NWTFQ&x-amz-security-token=FQoDYXdzEJH//////////wEaD

[39] FishSource. Anchoveta, Southern Peru/Northern Chile [Internet]. 2017.

[40] Froese R, Pauly D. FishBase. In: World Wide Web electronic publication. version (04/2012). [Internet]. 2012. www.fishbase.org

[41] Christensen V, De la Puente S, Sueiro JC, Steenbeek J, Majluf P. Valuing seafood: The Peruvian fisheries sector. Mar Policy. 2014;44: 302–311. 10.1016/j.marpol.2013.09.022

[42] Cahuin SM, Cubillos LA, Ñiquen M, Escribano R. Climatic regimes and the recruitment rate of anchoveta, Engraulis ringens, off Peru. Estuar Coast Shelf Sci. 2009;84: 591–597. 10.1016/j.ecss.2009.07.027

[43] Bertrand A, Chaigneau A, Peraltilla S, Ledesma J, Graco M, Monetti F, et al Oxygen: A fundamental property regulating pelagic ecosystem structure in the coastal southeastern tropical pacific. PLoS One. 2011;6: 2–9. 10.1371/journal.pone.0029558 22216315 10.1371/journal.pone.0029558 PMC3247266

[44] Schaefer MB. Some considerations of population dynamics and economics in relation to the management of the commercial marine fisheries. J Fish Res Board Canada. 1957;14: 669–681.

[45] Majluf P, De la Puente S, Christensen V. The little fish that can feed the world. Fish Fish. 2017;18: 772–777. 10.1111/faf.12206

[46] Froese R, Branch TA, Proelß A, Quaas M, Sainsbury K, Zimmermann C. Generic harvest control rules for European fisheries. Fish Fish. 2011;12: 340–351. 10.1111/j.1467-2979.2010.00387.x

[47] SEDAR. SEDAR 40 –Atlantic Menhaden Stock Assessment Report. Charleston, South Carolina; 2015.

[48] VanderKooy SJ, Smith J. The menhaden fishery of the Gulf of Mexico, United States: A regional management plan– 2015 revision. Oceans Springs, Mississippi; 2015.

[49] Omega Protein Corporation. Form 10-K. 2015.

[50] NMFS. Commercial Fisheries—Annual Landings [Internet]. 2017. http://www.st.nmfs.noaa.gov/commercial-fisheries/commercial-landings/annual-landings/index

[51] Harrington JM, Myers RA, Rosenberg AA. Wasted Resources: Bycatch and discards in U.S. Fisheries. 2005.

[52] Henry KA. Atlantic Menhaden (Brevoortia tyrannus). Resource and Fishery-Analysis of Decline. Seattle; 1971.

[53] Daybrook fisheries. Welcome to Daybrook Fisheries, INC. 2016; http://oceana.co.za/wp-content/uploads/2016/09/DaybrookPosters.pdf

[54] Friend of the Sea. Omega Protein Inc. FAO Area 31, Purse seine, Brevoortia petronus [Internet]. 2016. http://www.friendofthesea.org/public/catalogo/2017%2fOmega%2fProtein%2fWild%2freport.pdf

[55] Friend of the Sea. Omega Protein Inc. FAO Area 21, Purse seine, Brevoortia tyrannus [Internet]. 2016. http://www.friendofthesea.org/public/catalogo/2017%2fOmega%2fProtein%2fWild%2freport.pdf

[56] Kirkley JE. An Assessment of the Social and Economic Importance Of Menhaden (Brevoortia tyrannus) (Latrobe, 1802) in Chesapeake Bay Region. VIMS Marine Resource Report No. 2011–14. Gloucester Point, VA. Gloucester Point; 2011.

[57] NMFS. Commercial Landings—Data Caveats [Internet]. 2017. http://www.st.nmfs.noaa.gov/commercial-fisheries/commercial-landings/data-caveats/index

[58] Daybrook Fisheries; Gulf Menhaden Fish Oil & Fishmeal [Internet]. 2016. http://oceana.co.za/wp-content/uploads/2016/09/BookletFinal_2014.pdf

[59] Gutiérrez NL, Valencia SR, Branch TA, Agnew DJ, Baum JK, Bianchi PL, et al Eco-label conveys reliable information on fish stock health to seafood consumers. PLoS One. 2012;7: 1–8. 10.1371/journal.pone.0043765 22928029 10.1371/journal.pone.0043765 PMC3424161

[60] Sogn-Grundvag G, Larsen TA, Young JA. The value of line-caught and other attributes: An exploration of price premiums for chilled fish in UK supermarkets. Mar Policy. 2013;38: 41–44. 10.1016/j.marpol.2012.05.017

[61] Wilen JE. Stranded Capital in Fisheries: The Pacific Coast Groundfish / Whiting Case. Mar Resour Econ. 2009;24: 1–18.

[62] Pikitch EK, Rountos KJ, Essington TE, Santora C, Pauly D, Watson R, et al The global contribution of forage fish to marine fisheries and ecosystems. Fish Fish. 2014;15: 43–64. 10.1111/faf.12004

[63] Sumaila UR, Khan AS, Dyck AJ, Watson R, Munro G, Tydemers P, et al A bottom-up re-estimation of global fisheries subsidies. J Bioeconomics. 2010;12: 201–225. 10.1007/s10818-010-9091-8

[64] Sumaila UR, Lam V, Le Manach F, Swartz W, Pauly D. Global fisheries subsidies: An updated estimate. Mar Policy. 2016;69: 189–193. 10.1016/j.marpol.2015.12.026

[65] Merino G, Barange M, Mullon C, Rodwell L. Impacts of global environmental change and aquaculture expansion on marine ecosystems. Glob Environ Chang. 2010;20: 586–596. 10.1016/j.gloenvcha.2010.07.008

[66] Cubillos LA, Arcos DF. Recruitment of common sardine (Strangomera bentincki) and anchovy (Engraulis ringens) off central-south Chile in the 1990s and the impact of the 1997–1998 El Niño. Aquat Living Resour. 2002;15: 87–94. 10.1016/S0990-7440(02)01158-0

[67] Aranda M. Developments on fisheries management in Peru: The new individual vessel quota system for the anchoveta fishery. Fish Res. Elsevier; 2009;96: 308–312. 10.1016/J.FISHRES.2008.11.004

[68] Walters CJ, Pearse PH. Stock information requirements for quota management systems in commercial fisheries. Rev Fish Biol Fish. 1996;6: 21–42. 10.1007/BF00058518

[69] FIS. SNP estimates capture of 5 million tonnes of anchovy this year. In: FIS—World News [Internet]. 2017 [cited 12 Oct 2017]. http://www.fis.com/fis/worldnews/worldnews.asp?l=e&id=91308&ndb=1

[70] Larkin PA. An Epitaph for the Concept of Maximum Sustained Yield. Trans Am Fish Soc. 1977;106: 1–11. 10.1577/1548-8659(1977)106<1:AEFTCO>2.0.CO;2

[71] Merino G, Barange M, Blanchard JL, Harle J, Holmes R, Allen I, et al Can marine fisheries and aquaculture meet fish demand from a growing human population in a changing climate? Glob Environ Chang. Elsevier Ltd; 2012;22: 795–806. 10.1016/j.gloenvcha.2012.03.003

[72] Schueller A. GDAR 02—Gulf Menhaden Stock Assessment. Oceans Springs, Mississippi; 2016.

[73] NOAA. Forecast for the 2017 Gulf and Atlantic Menhaden Purse-Seine Fisheries and Review of the 2016 Fishing Season. Beaufort, NC; 2017.

[74] Pauly D. Anecdotes and the shifting baseline syndrome of fisheries. Trends Ecol Evol. 1995;10: 430 10.1016/S0169-5347(00)89171-5 21237093 10.1016/s0169-5347(00)89171-5

[75] Uphoff JH. Predator-prey analysis of striped bass and Atlantic menhaden in upper Chesapeake Bay. Fish Manag Ecol. 2003;10: 313–322. 10.1046/j.1365-2400.2003.00366.x

[76] Gordon H. S. The Economic Theory of a Common-Property Resource: The Fishery. J Polit Econ. 1954;62: 124–142.

