# Supplementary material for: Establishing company level fishing revenue and profit losses from fisheries: A bottom-up approach
Source: PLoS One. 2018 Nov 20;13(11):e0207768. doi: 10.1371/journal.pone.0207768 (PMC6245793; doi:10.1371/journal.pone.0207768)
Supplement: S1 Table — (DOCX) [file pone.0207768.s001.docx]

Table S1. Cost of fishing and ex-vessel prices by fishery (USD·tonne^-1^).

| Species | Cost of fishing | Ex-vessel price |
| --- | --- | --- |
| Anchoveta | 100.48^1^ | 133.50^1^ |
| Atlantic menhaden | 177.57^2^ | 204.31^3^ |
| Gulf menhaden | 177.57^2^ | 256.83^3^ |

1. [1]
2. [2] Assumed same cost of fishing for Gulf menhaden as Atlantic menhaden
3. [3]
4. Note: This does not include the elasticity adjusted prices based on increases in landings.

1. Christensen V, De la Puente S, Sueiro JC, Steenbeek J, Majluf P. Valuing seafood: The Peruvian fisheries sector. Mar Policy. 2014;44: 302–311. doi:10.1016/j.marpol.2013.09.022

2. Kirkley JE. An Assessment of the Social and Economic Importance Of Menhaden (Brevoortia tyrannus) (Latrobe, 1802) in Chesapeake Bay Region. VIMS Marine Resource Report No. 2011-14. Gloucester Point, VA. Gloucester Point; 2011.

3. NMFS. Commercial Fisheries - Annual Landings [Internet]. 2017. Available: http://www.st.nmfs.noaa.gov/commercial-fisheries/commercial-landings/annual-landings/index
