# Supplementary material for: Establishing company level fishing revenue and profit losses from fisheries: A bottom-up approach
Source: PLoS One. 2018 Nov 20;13(11):e0207768. doi: 10.1371/journal.pone.0207768 (PMC6245793; doi:10.1371/journal.pone.0207768)
Supplement: S2 Table — (DOCX) [file pone.0207768.s002.docx]

Table S2. Scenario analysis for north-central and southern anchoveta stocks of Peru

|  | North-Central | | North-Central | | North-Central | | South^1^ | | South^1^ | | South^1^ | |
| --- | --- | --- | --- | --- | --- | --- | --- | --- | --- | --- | --- | --- |
| Scenario (Biomass)^2^ | Baseline | | Modeled (807)^2^ | | PGY (807)^2^ | | Baseline | | Modeled (0)^2^ | | PGY (0)^2^ | |
| Years | Biomass | Landings | Biomass | Landings | Biomass | Landings | Biomass | Landings | Biomass | Landings | Biomass | Landings |
| 2000 | 9,086 | 9,110 | 9,086 | 5,365 | 9,086 | 4,970 | 2,777 | 606 | 2,777 | 636 | 2,777 | 551 |
| 2001 | 10,951 | 5,960 | 9,975 | 5,940 | 10,370 | 5,713 | 3,108 | 511 | 2,871 | 787 | 3,094 | 726 |
| 2002 | 10,628 | 6,720 | 15,042 | 9,224 | 13,214 | 5,713 | 3,347 | 1,844 | 3,054 | 1,093 | 3,405 | 1,046 |
| 2003 | 9,938 | 5,130 | 9,802 | 5,828 | 12,476 | 5,713 | 3,192 | 261 | 2,746 | 501 | 3,132 | 483 |
| 2004 | 10,704 | 8,070 | 10,060 | 5,995 | 12,347 | 5,713 | 3,327 | 991 | 3,816 | 879 | 4,254 | 728 |
| 2005 | 10,606 | 7,570 | 9,956 | 5,928 | 12,090 | 5,713 | 3,044 | 1,428 | 2,788 | 1,009 | 3,442 | 959 |
| 2006 | 9,681 | 5,000 | 12,083 | 7,306 | 13,943 | 5,713 | 2,926 | 1,161 | 3,038 | 1,205 | 3,715 | 1,051 |
| 2007 | 8,737 | 5,140 | 11,072 | 5,357 | 13,643 | 5,713 | 2,532 | 1,259 | 3,473 | 1,153 | 4,216 | 980 |
| 2008 | 8,654 | 5,300 | 9,689 | 5,106 | 11,311 | 5,713 | 2,161 | 1,134 | 2,912 | 1,115 | 3,713 | 1,039 |
| 2009 | 8,445 | 5,260 | 9,689 | 5,149 | 10,561 | 5,713 | 1,983 | 740 | 2,913 | 928 | 3,699 | 883 |
| 2010 | 8,195 | 3,020 | 9,690 | 5,093 | 9,957 | 5,713 | 1,805 | 403 | 2,913 | 507 | 3,658 | 625 |
| 2011 | 8,996 | 6,310 | 9,690 | 4,037 | 9,334 | 5,256 | 1,915 | 881 | 2,912 | 649 | 3,266 | 663 |
| 2012 | 9,031 | 3,330 | 9,568 | 5,676 | 7,988 | 3,656 | 1,944 | 483 | 2,678 | 529 | 2,978 | 524 |
| 2013 | 9,289 | 4,500 | 10,267 | 4,170 | 10,512 | 5,713 | 1,947 | 332 | 3,495 | 496 | 3,826 | 376 |
| 2014 | 7,907 | 1,920 | 9,689 | 3,122 | 8,378 | 4,113 | 1,768 | 451 | 2,979 | 559 | 3,726 | 498 |
| 2015 | 8,332 | 3,340 | 9,690 | 3,785 | 7,332 | 2,913 | 1,793 | 396 | 2,564 | 582 | 3,403 | 602 |
| Mean (10^3^ t) | 9,324 | 5,355 | 10,315 | 5,443 | 10,784 | 5,234 | 2,473 | 805 | 2,996 | 789 | 3,519 | 733 |
| Coefficient of Variation (%) | 10.5 | 36.0 | 13.9 | 26.3 | 19.0 | 16.9 | 24.8 | 57.0 | 11.0 | 33.3 | 11.7 | 31.2 |
| 95% CI (10^3^ t) | 481.5 | 945.9 | 704.8 | 702.4 | 1002.9 | 433.1 | 300.8 | 224.8 | 162.0 | 128.7 | 202.4 | 112.0 |

1. Note: Catches can exceed biomass in certain years in the baseline scenario due to a high recruitment anomaly, and a lack of intra-year measurement.
2. The number in brackets refers to the biomass (10^3^ t) reference point, i.e. the biomass not subject to fishing mortality each year for the modeled scenario as established based on the scenario analysis methods.
