# Supplementary material for: Establishing company level fishing revenue and profit losses from fisheries: A bottom-up approach
Source: PLoS One. 2018 Nov 20;13(11):e0207768. doi: 10.1371/journal.pone.0207768 (PMC6245793; doi:10.1371/journal.pone.0207768)
Supplement: S3 Table — (DOCX) [file pone.0207768.s003.docx]

Table S3. Landings (10^3^ t) by company by year for the Peruvian anchoveta fishery based on quota ownership.

| Company | 2011 | 2012 | 2013 | 2014 | 2015 | Average |
| --- | --- | --- | --- | --- | --- | --- |
| Tecnologica De Alimentos S.A. | 1011 | 534 | 681 | 331 | 524 | 616 |
| Corporacion Pesquera Inca S.A.C. | 699 | 369 | 491 | 217 | 368 | 429 |
| Pesquera Diamante S.A. | 595 | 314 | 404 | 192 | 309 | 363 |
| Austral Group S.A.A | 460 | 243 | 319 | 145 | 241 | 282 |
| Pesquera Exalmar S.A.A. | 452 | 239 | 312 | 144 | 236 | 277 |
| Pesquera Hayduk S.A. | 428 | 226 | 299 | 134 | 225 | 262 |
| CFG Investment S.A.C. | 462 | 244 | 303 | 156 | 237 | 280 |

1. We present Corporacion Pesquera Inca S.A.C. and CFG Investment S.A.C. separately here, as they were not under the same ownership for the entire period.
