# Supplementary material for: Establishing company level fishing revenue and profit losses from fisheries: A bottom-up approach
Source: PLoS One. 2018 Nov 20;13(11):e0207768. doi: 10.1371/journal.pone.0207768 (PMC6245793; doi:10.1371/journal.pone.0207768)
Supplement: S4 Table — (DOCX) [file pone.0207768.s004.docx]

Table S4. Average attained landings for the top seven companies for 2011-2015 (10^3^ t), modeled higher landings (10^3^ t) based on Optimal F, and their impact on revenue and profits (USD 10^6^).

| Company | Landings^1^ | Optimal F landings^1,2^ | Revenue shortfall | Cost of landings shortfall | Unrealized profits |
| --- | --- | --- | --- | --- | --- |
| Tecnologica de Alimentos S.A. | 617 | 700 | 10.99 | 8.36 | 2.64 |
| Corporacion Pesquera Inca S.A.C. | 398 | 435 | 4.82 | 3.65 | 1.17 |
| Pesquera Diamante S.A. | 368 | 414 | 6.04 | 4.59 | 1.45 |
| Austral Group S.A.A. | 287 | 319 | 4.27 | 3.24 | 1.03 |
| Pesquera Exalmar S.A.A. | 221 | 243 | 2.93 | 2.22 | 0.71 |
| Pesquera Hayduk S.A. | 273 | 302 | 3.82 | 2.90 | 0.92 |
| CFG Investment S.A.C. | 252 | 291 | 5.09 | 3.87 | 1.21 |

1. Company level landings are from vessel level landings provided by [1]. Optimal F landings here are not based on company quota but on average share of landings from 2011-2015.
2. Optimal-F landings are generated by optimizing the biomass reference point, and fishing at F_MSY_ when B ≥_B_MSY_.

1. PRODUCE. Embarcaciones Pesqueras [Internet]. 2017 [cited 27 Jul 2017]. Available: http://www.produce.gob.pe/index.php/shortcode/servicios-pesca/embarcaciones-pesqueras
