# Supplementary material for: Establishing company level fishing revenue and profit losses from fisheries: A bottom-up approach
Source: PLoS One. 2018 Nov 20;13(11):e0207768. doi: 10.1371/journal.pone.0207768 (PMC6245793; doi:10.1371/journal.pone.0207768)
Supplement: S5 Table — (DOCX) [file pone.0207768.s005.docx]

Table S5. Estimated landings (10^3^ t) by company for the US menhaden fishery.

| Company | Menhaden | 2012 | 2013 | 2014 | 2015 | 2016 | Average |
| --- | --- | --- | --- | --- | --- | --- | --- |
| Omega Protein | Gulf | 242 | 214 | 187 | 262 | 235 | 225 |
| Omega Protein | Atlantic | 173 | 129 | 134 | 155 | 144 | 148 |
| Daybrook Fisheries | Gulf | 200 | 176 | 154 | 216 | 194 | 186 |
