# Supplementary material for: Establishing company level fishing revenue and profit losses from fisheries: A bottom-up approach
Source: PLoS One. 2018 Nov 20;13(11):e0207768. doi: 10.1371/journal.pone.0207768 (PMC6245793; doi:10.1371/journal.pone.0207768)
Supplement: S1 Appendix — (DOCX) [file pone.0207768.s006.docx]

# **S1 Appendix. Supplemental Methods**

The final step of estimating fisheries revenues and profits is subject to additional uncertainty, as prices change over time. To account for this, we created a multivariate log-transformed linear regression model to measure the ex-vessel price elasticity of menhaden to increased landings. We compiled monthly data on menhaden landings, menhaden ex-vessel prices and fishmeal world prices for 1990-2016 [1,2]. We log transformed all variables to properly account for their distribution. In addition, when the model is log transformed on both sides (i.e., a log-log model), the estimated coefficients are equivalent to the elasticity of the independent variable, and this elasticity is constant [3]. The equation estimated was

ln(P_t_) = β_0_ + β_1_ln(L_t_) + β_2_ln(FM_t_) + ε_t_

Where P is the ex-vessel price of menhaden, L is the amount of landings, FM is the price of fishmeal, and t is the time period (month and year), and ε is the error term. As we only had monthly price and landings data for menhaden, we assumed the price elasticity to be the same for the anchoveta fishery.

The multivariate linear regression model of menhaden ex-vessel prices had an adjusted R^2^ of 0.685, suggesting a reasonable fit to the data. The coefficient we were concerned with in this study, i.e., log(Landings), was -0.075 and was statistically significant (p < 2e-16). This coefficient was equal to the elasticity effect on price and can therefore be interpreted as a 1% increase in landings will result in a 0.075% decrease in ex-vessel prices. This price-elasticity estimate was applied to both anchoveta and menhaden ex-vessel prices when landings increased in our modeled scenarios.

# **Supplementary Material References**

1. NMFS. Commercial Fisheries - Monthly Landings [Internet]. 2017 [cited 5 Apr 2017]. Available: http://www.st.nmfs.noaa.gov/commercial-fisheries/commercial-landings/annual-landings/index

2. World Bank. GEM Commodities [Internet]. Available: https://datacatalog.worldbank.org/dataset/gem-commodities

3. Hill RC, Griffiths WE, Lim GC. Principles of Econometrics. 4th Editio. Hoboken: John Wiley & Sons; 2011.
